# Nifedipine Increases Iron Content in WKPT-0293 Cl.2 Cells via Up-Regulating Iron Influx Proteins

**DOI:** 10.3389/fphar.2017.00060

**Published:** 2017-02-13

**Authors:** Shuang-Shuang Yu, Li-Rong Jiang, Yan Ling, Zhong-Ming Qian, Yu-Fu Zhou, Juan Li, Ya Ke

**Affiliations:** ^1^Laboratory of Neuropharmacology, Fudan University School of PharmacyPudong, China; ^2^School of Biomedical Sciences, Faculty of Medicine, The Chinese University of Hong KongHong Kong, Hong Kong

**Keywords:** nifedipine, iron transport proteins, WKPT-0293 Cl.2 cells, urinary iron, cell iron

## Abstract

Nifedipine was reported to enhance urinary iron excretion in iron overloaded mice. However, it remains unknown how nifedipine stimulates urinary iron excretion in the kidney. We speculated that nifedipine might inhibit the TfR1/ DMT1 (transferrin receptor 1/divalent metal transporter1)-mediated iron uptake by proximal tubule cells in addition to blocking L-type Ca2+ channels, leading to an increase in iron in lumen-fluid and then urinary iron excretion. To test this hypothesis, we investigated the effects of nifedipine on iron content and expression of TfR1, DMT1 and ferroportin1 (Fpn1) in WKPT-0293 Cl.2 cells of the S1 segment of the proximal tubule in rats, using a graphite furnace atomic absorption spectrophotometer and Western blot analysis, respectively. We demonstrated for the first time that nifedipine significantly enhanced iron content as well as TfR1 and DMT1 expression and had no effect on Fpn1 levels in the cells. We also found that ferric ammonium citrate decreased TfR1 levels, increased Fpn1 expression and had no effect on DMT1 content, while co-treatment with nifedipine and FAC increase TfR1 and DMT1 expression and also had no effect on Fpn1 levels. These findings suggest that the nifedipine-induced increase in cell iron may mainly be due to the corresponding increase in TfR1 and DMT1 expression and also imply that the effects of nifedipine on iron transport in proximal tubule cells can not explain the increase in urinary iron excretion.

## Introduction

Plasma iron is tightly bound to Tf, a 78-kDa circulating protein that is assumed to not cross the glomerulus filter, because of its low protein permeability (Aisen, 1994; Linder, 2013). The kidney has therefore been regarded as more or less irrelevant in terms of iron handling, and for a long time, iron metabolism in the kidney also received relatively little attention (Smith and Thévenod, 2009; Thévenod and Wolff, 2016). However, a number of recent studies have provided evidence for the presence of many, if not all of the iron transporters and receptors in renal tubular cells, including TfR1 (Zhang et al., 2007; Moulouel et al., 2013), cubilin (a novel Tf receptor) (Kozyraki et al., 2001), divalent metal transporter 1 (DMT1) (Canonne-Hergaux and Gros, 2002) at the apical membrane, and ferroportin 1 (Fpn1) (Wolff et al., 2011) at the basolateral membrane. Previous studies also have suggested that Tf might be able to filter from the glomerulus (Alfrey and Hammond, 1990; Norden et al., 2001; Kozyraki et al., 2001), and that transferrin-bound iron (Tf-fe) could be reabsorbed by proximal tubule cells via TfR1- or cubilin-mediated endocytosis (Kozyraki et al., 2001; Moulouel et al., 2013). Measurement of iron re-absorption in the rat kidney *in vivo* has shown that, under physiological conditions, about 0.4 mg iron is filtered daily and about 0.7% is excreted in the urine (Wareing et al., 2000). These novel data imply that the kidney plays a previously unsuspected role in systemic iron balance, and contributes to general iron homeostasis (Martines et al., 2013; Veuthey et al., 2014).

A recent study reported that L-type Ca2+ channel blockers of the dihydropyridine type, including nifedipine and niguldipine, markedly stimulated iron transport in both COS-7 and HEK293T cells, which were transiently transfected with DMT-1A with IRE, and enhanced liver iron mobilization as well as urinary iron excretion in animal models of iron overload (Ludwiczek et al., 2007). These results revealed a new pharmacological property of nifedipine and related compounds for the treatment of iron overloaded disorders by increasing excretion of iron from the kidney. However, it is unknown how nifedipine stimulates urinary iron excretion in the kidney. In the rat heart, nifedipine was demonstrated to be able to inhibit L-type Ca2+ channel-mediated non-transferrin-bound iron (NTBI) uptake by isolated hearts and ventricular myocytes (Tsushima et al., 1999). The presence of DMT1 has been well-documented in the heart (Ke et al., 2003; Zhao et al., 2010). It has also been shown that DMT1 is a voltage-dependent divalent-cation transporter and NTBI uptake-mediated by DMT1 has similar voltage dependency to that of the L-type Ca2+ channel (Tsushima et al., 1999). Although it is currently undetermined whether L-type Ca2+ channel blockers affect DMT1 transport properties, it is possible that the nifedipine-induced reduction in iron uptake, as observed in isolated rat hearts and ventricular myocytes, is mediated by the inhibition of not only the L-type Ca2+ channel, but also DMT1.

Based on these novel findings, we therefore speculated that nifedipine might be able to inhibit TfR1/DMT1-mediated iron uptake in addition to blocking the L-type Ca2+ channels and their associated iron uptake, and then reduce the amount of iron transport across the cell membrane from tubular lumen-fluid to the inside of cells in the proximal tubule (an important segment of physiological re-absorption), leading to an increase in iron in the final urine. To test this hypothesis, in the present study we investigated the effects of nifedipine on iron content, as well as the expression of iron uptake proteins TfR1 and DMT1 and iron release protein Fpn1 in WKPT-0293 Cl.2 cells of the S1 segment of the proximal tubule of normotensive Wistar-Kyoto rats (RPTC).

## Materials and Methods

### Materials

Unless otherwise stated, all chemicals, including MTT and mouse monoclonal anti-β-actin, were obtained from the Sigma Chemical Company, St. Louis, MO, USA. The mouse anti-rat TfR1 antibody was purchased from Invitrogen, Carlsbad, CA, USA, and the rabbit polyclonal anti-mouse Fpn1 antibody from Chemicon International, Temecula, CA, USA. The rabbit anti-rat DMT1 + IRE and DMT1 - IRE polyclonal antibodies were bought from the Alpha Diagnostic International Company, San Antonio, TX, USA, and goat anti-rabbit or anti-mouse IRDye 800 CW secondary antibodies from LI-COR Biosciences, Lincoln, NE, USA. Dulbecco’s Modified Eagle’s Medium (DMEM)/Ham’s F-12 was obtained from GIBCO, Grand Island, NY, USA and BSA from the Calbiochem-Novabiochem Corporation, San Diego, CA, USA. The scintillation cocktail and tubes were obtained from the Beckman Coulter Company, Fullerton, CA, USA. All experimental protocols were performed according to the Animal Management Rules of the Ministry of Health of China, and approved by the Animal Ethics Committees of Fudan University and The Chinese University of Hong Kong.

### Cell Culture

Immortalized cells (WKPT-0293 Cl.2) from the S1 segment of the proximal tubule of normotensive Wistar-Kyoto rats (RPTC) were cultured as previously described Woost et al. (1996). Briefly, cells were maintained in renal tubular epithelium medium composed of DMEM and F-12 [nutrient mixture F-12 (Ham)] at a ratio of 1:1, supplemented with 15 mM HEPES, 1.2 mg/ml NaHCO_3_, 5 μg/ml Tf, 10 ng/ml epidermal growth factor, 100 U/ml penicillin G, 100 mg/ml streptomycin sulfate, and 10% fetal calf serum. Cells were plated at a density of 5 × 10^4^/ml on collagen-coated flasks, passaged at 80% confluency, and split 1:10, twice a week.

### Assessment of Cell Viability

Cell viability was assessed using an MTT assay as previously described (He et al., 2008; Du et al., 2009). Briefly, a total of 25 μl MTT (1 g/L in PBS) was added to each well before incubation was conducted at 37°C for 4 h. The assay was stopped by the addition of a 100 μl lysis buffer (20% SDS in 50% N’Ndimethylformamide, pH 4.7). Optical density (OD) was measured at the 570 nm wavelength by the use of an ELX-800 microplate assay reader (Bio-tek, Winooski, VT, USA) and the results were expressed as a percentage of the absorbance measured in the control cells.

### Graphite Furnace Atomic Absorption Spectrophotometer

Total iron in the WKPT-0293 Cl.2 cells was determined using a graphite furnace atomic absorption spectrophotometer (GFAAS, Perkin Elmer, Analyst 100) as previously described (Chang et al., 2005; Ke et al., 2005). The cells were diluted 1:20 (wt/v) with HEPES buffer and homogenized with a sonicator (MSE Soniprep 150 Ultrasonic Disintegrator, MSE Scientific Instruments, England). A 50-μl portion of the homogenate was added to an equal volume of ultra-pure nitric acid in a 0.5 ml polypropylene microfuge tube, digested for 48 h at 50°C, and diluted 1:40 with 3.12 mmol/L nitric acid for iron analysis. Standard curves (0–40 ppb) were prepared by diluting iron standard with blanks prepared from homogenization reagents in 0.2% HNO3. Standards and digested samples were read in triplicate by injecting 50 μl aliquots including 0.05 mg Mg(NO_3_)_2_ as matrix modification into graphite furnace. Absorbance readings at 248.3 nm, slit at 0.2 nm, pretreatment temperature at 1400°C, atomization temperature at 2400°C were recorded.

### Western Blot Analysis

WKPT-0293 Cl.2 cells that received different treatments were washed with ice-cold PBS, homogenized with lysis buffer and then subjected to sonication using a Soniprep 150 (MSE Scientific Instruments, London, UK). After centrifugation at 10,000 *g* for 15 min at 4°C, the supernatant was collected and protein content was determined using the Bradford assay kit (Bio-Rad). Aliquots of the cell extract containing 50 μg of protein were diluted in 2 μl of sample buffer (50 mM Tris, pH 6.8, 2% SDS, 10% glycerol, 0.1% bromphenol blue, and 5% β-mercaptoethanol) and heated for 5 min at 95°C before SDS-PAGE on 10% gel and transfer to a pure nitrocellulose membrane. After the transfer, the membrane was blocked with 5% blocking reagent in Tris-buffered saline containing 0.1% Tween-20, overnight at 4°C. The membrane was then rinsed in three changes of Tris-buffered saline/Tween-20, incubated in fresh washing buffer once for 15 min and twice for 5 min, and then incubated overnight at 4°C with primary antibodies: rabbit anti-rat DMT1 + IRE, DMT1-IRE polyclonal antibodies, rabbit anti-mouse Fpn1 polyclonal antibody (1:5000), and mouse anti-rat TfR1 monoclonal antibody (1:1000). After three washes, the blots were incubated with goat anti-rabbit or anti-mouse IRDye 800 CW secondary antibody (1:5000, Li-Cor) for 1 h at room temperature. The intensity of the specific bands was detected and analyzed by the Odyssey infrared imaging system (Li-Cor) (Du et al., 2014; Qian et al., 2014). To ensure even loading of the samples, the same membrane was probed with mouse monoclonal anti-β-actin antibody at a 1:2000 dilution.

### Statistical Analysis

Statistical analyses were performed using Graphpad Prism. Data were presented as mean ± SEM. The differences between the means were all determined by two-way analysis of variance (ANOVA). A probability value of *P* < 0.05 was taken to be statistically significant.

## Results

### Effects of Nifedipine and Ferric Ammonium Citrate (FAC) Treatment on the Viability of WKPT-0293 Cl.2 Cells

**FIGURE 1:**
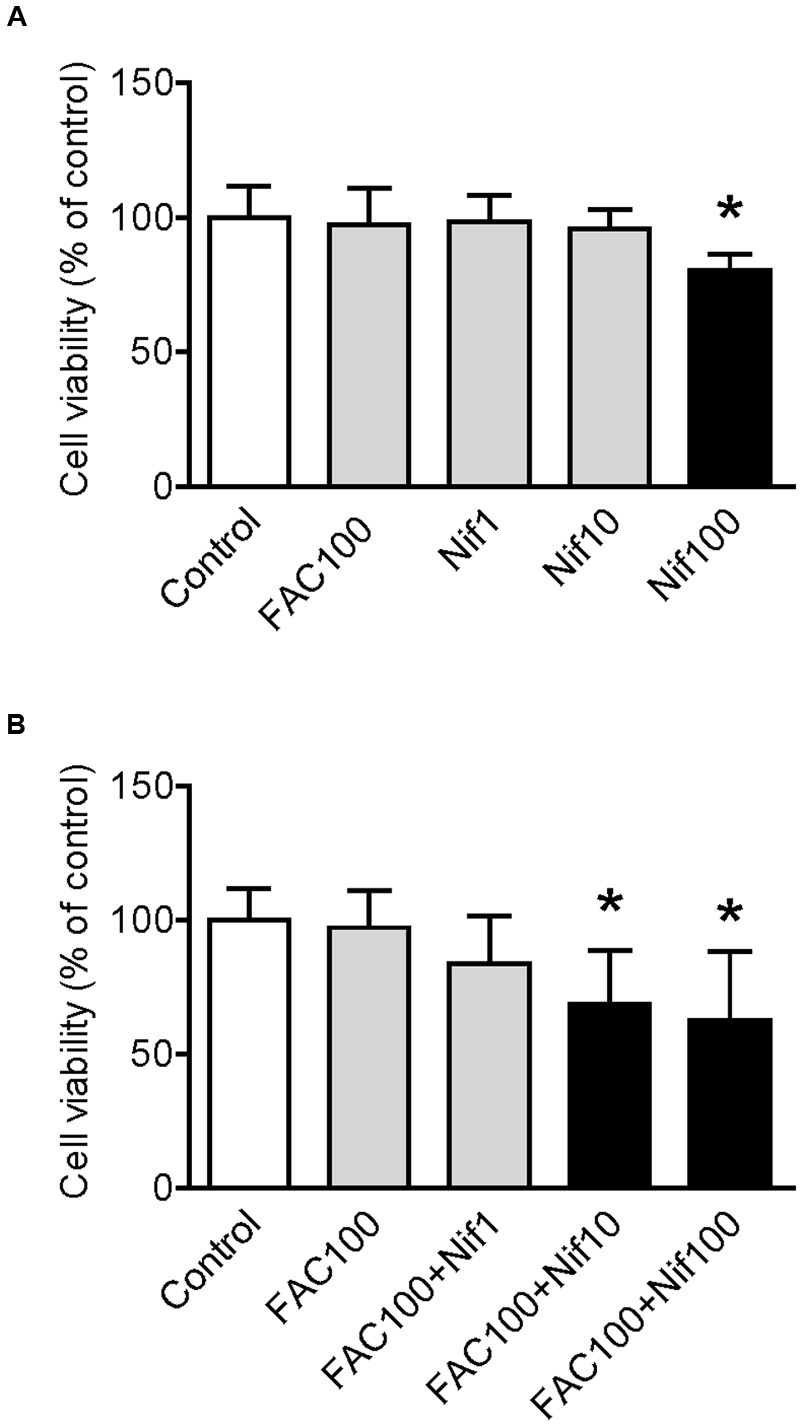
**Effect of nifedipine and FAC treatment on the viability of WKPT-0293 Cl.2 cells.** WKPT-0293 Cl.2 cells were treated with FAC (100 μM) or nifedipine (1, 10, 100 μM) alone for 16 h **(A)**; or FAC (100 μM) with nifedipine (1, 10, 100 μM) for 16 h **(B)**, cell viability was then evaluated using the MTT assay as described in the Section “Materials and Methods.” Data were presented as means ± SEM (*n* = 8). ^∗^*p* < 0.05 versus the control.

We first investigated the effects of FAC and nifedipine on the viability of WKPT-0293 Cl.2 by treating the cells with FAC (100 μM) (Chen et al., 2005) or various concentrations (1, 10, 100 μM) of nifedipine alone for 16 h, and then evaluated cell viability using an MTT assay. The results showed that there were no significant differences in viability between the control cells and the cells treated with 100 μM of FAC or 1 and 10 μM of nifedipine. However, viability in the cells treated with 100 μM of nifedipine was found to be significantly lower than that of the control cells (**Figure 1A**).

To find out the effects of co-treatment with FAC and nifedipine on the viability of WKPT-0293 Cl.2 cells, the cells were treated with FAC (100 μM) plus 1, 10 or 100 μM of nifedipine for 16 h. The MTT assay demonstrated that viability in the cells treated with 1 μM of nifedipine plus FAC or FAC alone were not significantly different from that of the control cells, but treatment with 10 or 100 μM of nifedipine plus FAC did induce a significant reduction in cell viability (**Figure 1B**). The findings here demonstrated that nifedipine could promote the toxic effects of FAC on the viability of WKPT-0293 Cl.2 cells.

### Nifedipine Treatment Increased Iron Level in WKPT-0293 Cl.2 Cells

**FIGURE 2:**
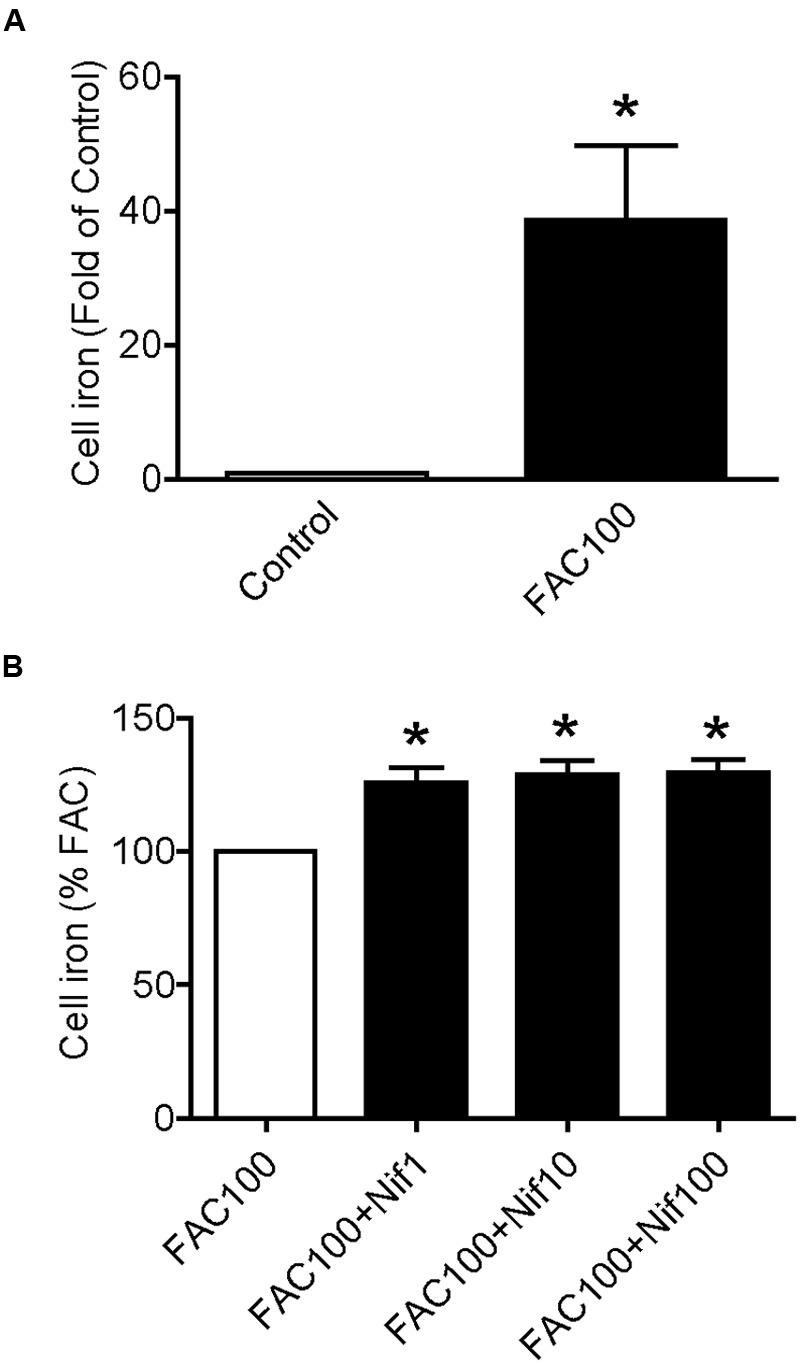
**Nifedipine treatment increased iron level in WKPT-0293 Cl.2 cells.** WKPT-0293 Cl.2 cells were treated with FAC (100 μM) **(A)** or nifedipine (1, 10, 100 μM) with FAC (100 μM) **(B)** for 16 h, iron content was then measured using a GFAAS (graphite furnace atomic absorption spectrophotometer) as described in the Section “Materials and Methods.” The data were presented as Mean ± SEM (*n* = 8). ^∗^*p* < 0.05, versus the control **(A)** or FAC **(B)**.

We then examined the effects of nifedipine on iron uptake in WKPT-0293 Cl.2 cells. The cells were treated with FAC (100 μM) with different concentrations (0, 1, 10, 100 μM) of nifedipine for 16 h, and iron content was then measured using a GFAAS. It was found that treatment with 100 μM of FAC alone induced a significant increase in iron content, 38.629 fold of that of the control cells (**Figure 2A**). The addition of nifedipine led to a further increase in cell iron levels. Iron contents in the cells treated with 1, 10, or 100 μM of nifedipine plus 100 μM of FAC were significantly higher than those in the cells treated with 100 μM of FAC alone. There were no significant differences between the cells treated with different concentrations of nifedipine plus FAC (**Figure 2B**). These results showed that nifedipine has a role in increasing the iron uptake of WKPT-0293 Cl.2 cells.

### Nifedipine Treatment Increased Expression of TfR1, DMT1 + IRE and DMT1 - IRE in WKPT-0293 Cl.2 Cells

**FIGURE 3:**
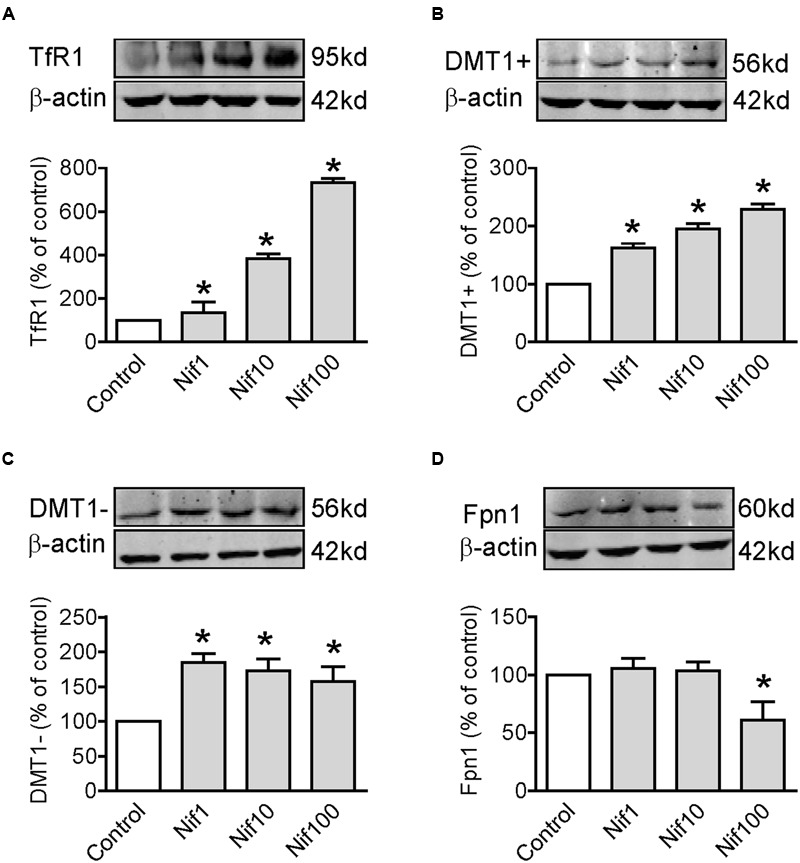
**Nifedipine treatment increased expression of TfR1, DMT1 + IRE and DMT1 - IRE in WKPT-0293 Cl.2 cells.** WKPT-0293 Cl.2 cells were treated with nifedipine (1, 10, 100 μM) for 16 h, and the expression of TfR1 **(A)**, DMT1 + IRE **(B)**, DMT1 + IRE **(C)**, and Fpn1 **(D)** was then measured by Western blot analysis. The data were presented as Mean ± SEM (*n* = 6, % Control). ^∗^*p* < 0.05 versus the control.

To find out the potential mechanisms involved in the positive role of nifedipine on iron uptake by WKPT-0293 Cl.2 cells, we investigated the effects of nifedipine on the expression of major iron uptake proteins TfR1, DMT1 + IRE and DMT1 - IRE, as well as iron release protein Fpn1 in the cells. We demonstrated that nifedipine induced a progressive increase in TfR1 (**Figure 3A**) and DMT1 + IRE (**Figure 3B**) expression but not in DMT1 - IRE and Fpn1 with the concentrations added. Although the expression of DMT1 - IRE in cells treated with 1, 10, or 100 μM of nifedipine was significantly higher than that of the control cells, there were no significant differences between cells treated with different concentrations of nifedipine (**Figure 3C**). Treatment with 1 or 10 μM of nifedipine did not induce any significant changes in Fpn1 expression, with no significant differences being found in Fpn1 content between the cells treated with 1 or 10 μM of nifedipine and the controls (**Figure 3D**). However, treatment with 100 μM of nifedipine was found to induce a significant reduction in Fpn1 expression in the WKPT-0293 Cl.2 cells. The data imply that nifedipine likely increases iron uptake in WKPT-0293 Cl.2 cells via its role in up-regulating expression of iron uptake proteins.

### FAC Treatment Decreased TfR1 and Increased Fpn1 Expression in WKPT-0293 Cl.2 Cells

**FIGURE 4:**
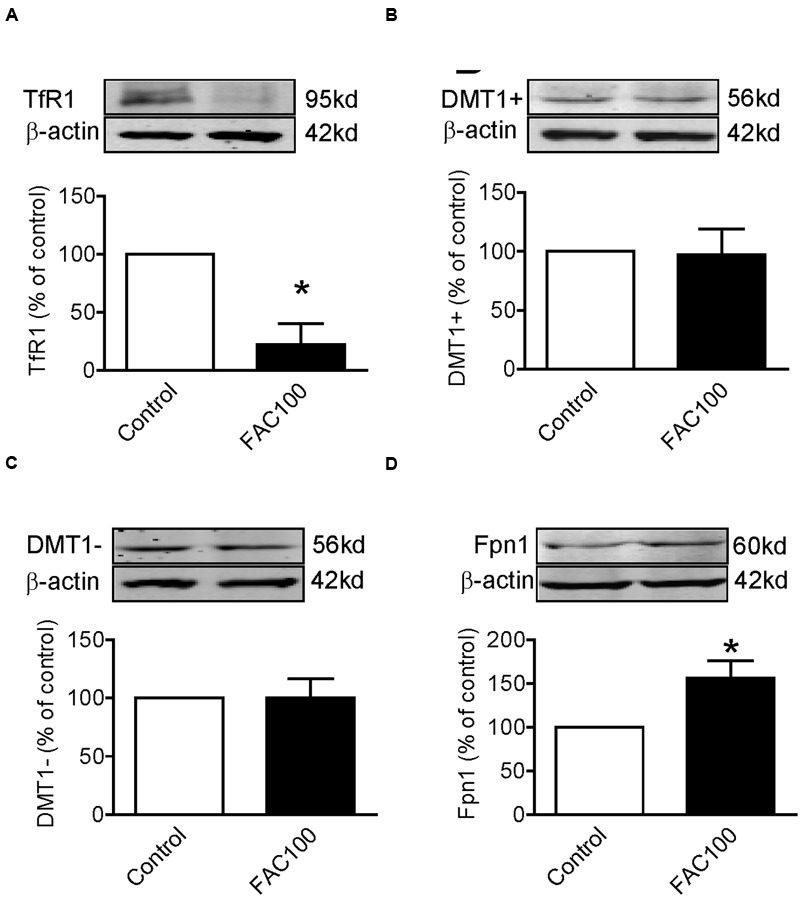
**Ferric ammonium citrate treatment decreased TfR1 and increased Fpn1 expression in WKPT-0293 Cl.2 cells.** WKPT-0293 Cl.2 cells were treated with FAC (100 μM) for 16 h, and the expression of TfR1 **(A)**, DMT1 + IRE **(B)**, DMT1 - IRE **(C)**, and Fpn1 **(D)** was then measured by Western blot analysis. The data were presented as Mean ± SEM (*n* = 6, % Control). ^∗^*p* < 0.05 versus the control.

We also investigated the effects of iron-overload on the expression of TfR1, DMT1 + IRE, DMT1 - IRE and Fpn1 by treating WKPT-0293 Cl.2 cells with 100 μM of FAC for 16 h. Western blot analysis showed that FAC treatment induced a significant reduction in TfR1 (**Figure 4A**) and an increase in Fpn1 (**Figure 4D**) expression, and had no effect on DMT1 + IRE (**Figure 4B**) and DMT1 - IRE (**Figure 4C**) expression in WKPT-0293 Cl.2 cells.

### Co-treatment With Nifedipine and FAC Increased TfR1, DMT1 + IRE and DMT1 - IRE Expression in WKPT-0293 Cl.2 Cells

**FIGURE 5:**
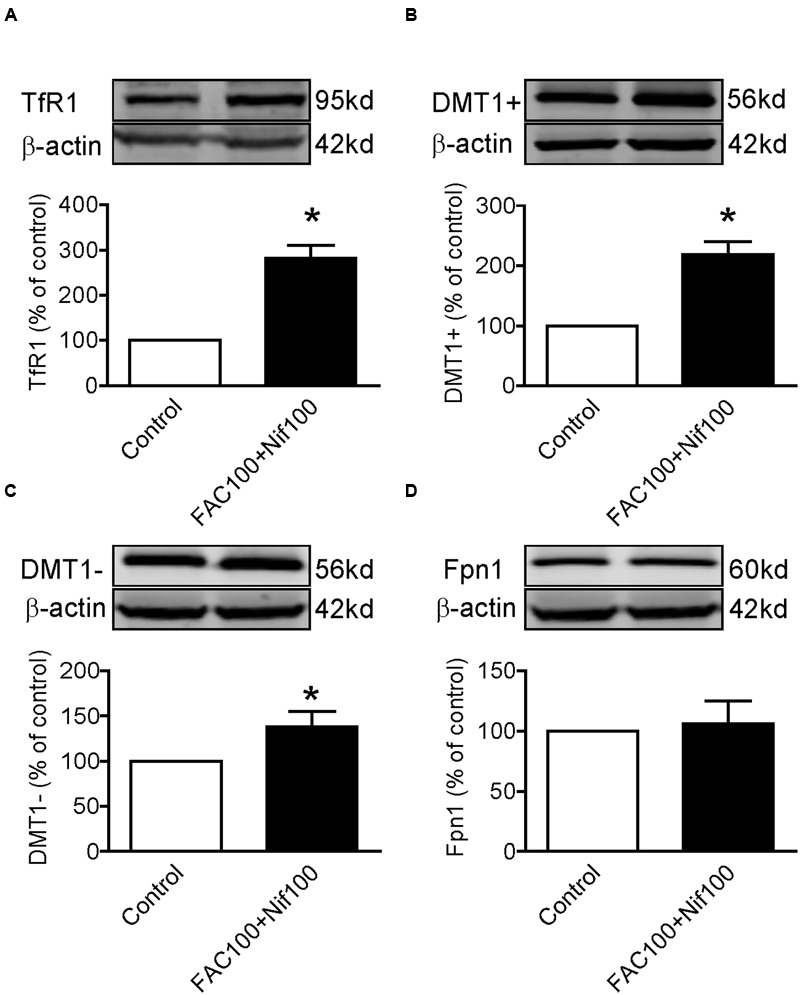
**Co-treatment with nifedipine and FAC increased TfR1, DMT1 + IRE and DMT1 - IRE expression in WKPT-0293 Cl.2 cells.** WKPT-0293 Cl.2 cells were treated with 100 μM of nifedipine plus100 μM of FAC for 16 h, and the expression of TfR1 **(A)**, DMT1 + IRE **(B)**, DMT1 - IRE **(C)**, and Fpn1 **(D)** was then measured by Western blot analysis. The data were presented as Mean ± SEM (*n* = 4, % Control). ^∗^*p* < 0.05 versus the control.

Finally, we investigated the effects of co-treatment with Nifedipine and FAC on the expression of TfR1, DMT1 + IRE, DMT1 - IRE and Fpn1 by treating WKPT-0293 Cl.2 cells with 100 μM of nifedipine plus100 μM of FAC for 16 h. Western blot analysis demonstrated that co-treatment with nifedipine and FAC led to a significant increase in TfR1 (**Figure 5A**), DMT1 + IRE (**Figure 5B**) and DMT1-IRE (**Figure 5C**) expression and had no effect on Fpn1 expression (**Figure 5D**) in WKPT-0293 Cl.2 cells.

## Discussion

The major objective of this study was to find out whether treatment with the L-type Ca2+ channel blocker nifedipine could reduce iron uptake by the proximal tubule cells of the nephron, leading to an increase in the iron content within luminal fluid and, subsequently to also an increase in urinary iron excretion in the kidney. We therefore first investigated the effect of nifedipine on iron content in proximal tubule cells treated with FAC. We demonstrated for the first time that nifedipine treatment has a significant role in enhancing, rather than reducing iron content in WKPT-0293 Cl.2 cells of the S1 segment of the proximal tubule of normotensive Wistar-Kyoto rats (RPTC). This unexpected finding, that is, that nifedipine induces an increase in cell iron content, should predictably lead to a decrease rather than an increase in urinary iron excretion. It implies that the effects of nifedipine on iron transport in proximal tubule cells are not associated with its induced increase in urinary iron excretion in the kidney.

**FIGURE 6:**
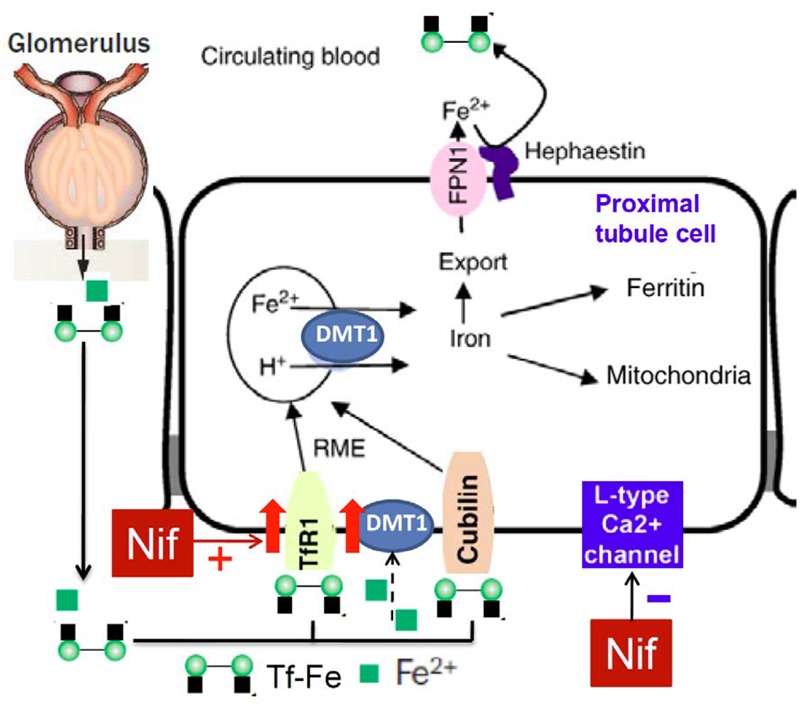
**Hypothetical scheme for the possible effects of nifedipine on TfR1, DMT1 and Fpn1 expression and iron re-absorption in proximal tubule cells of kidney**.

To find out the mechanisms involved in the nifedipine-induced increase in cell iron content, we further examined the effects of nifedipine and/or FAC on the expression of iron transport proteins TfR1, DMT1 + IRE, DMT1 - IRE, and Fpn1 respectively in WKPT-0293 Cl.2 cells. It was found that FAC alone decreased TfR1 expression, increased Fpn1 expression and had no effects on DMT1 + IRE and DMT1 - IRE, nifedipine alone increased TfR1, DMT1 + IRE and DMT1 - IRE expression, and had no effect on Fpn1, while co-treatment with nifedipine and FAC led to a significant increase in TfR1, DMT1 + IRE and DMT1 - IRE expression and also had no effect on Fpn1 expression in WKPT-0293 Cl.2 cells. These results implied that that the nifedipine-induced increase in iron content in the cells may mainly be due to the increased expression of TfR1 and DMT1 (**Figure 6**). FAC may be either donor of iron to trarnsferrin in the medium or as an NTBI source. Most of the iron taken up by the cells was probably in the form of Tf-Fe via a TfR1 (cell membrane)/ DMT1 (endosomal membrane) pathway because we did not deplete the intracellular store of Tf by suspending the cells in DMEM–HEPES medium for a given period (Chang et al., 2007; Qian et al., 2011) in this study. It is also conceivable, but remains to be tested, that small amount of the iron may be taken up by the cells in the form of NTBI via a DMT1-mediated pathway. In addition, further investigations on how nifedipine enhances TfR1 and DMT1 expression in this type of cells are needed. The relevant mechanisms, especially the effects of nifedipine on the expression of iron regulatory proteins (IRPs) and membrane potential polarization (Du et al., 2016) need to be clarified as well.

Studies in Belgarde rats have shown that DMT1, also known as DCT1 (Gunshin et al., 1997) or NRAMP2 (Fleming et al., 1997), plays a fundamental role in body iron homeostasis (Fleming et al., 1998, 1999; Garrick et al., 1999; Gruenheid et al., 1999). DMT1 is highly expressed in the kidney (Canonne-Hergaux and Gros, 2002) and is also suggested to play a role in urinary iron handling (Martines et al., 2013; Veuthey et al., 2014). Therefore, modulation of renal DMT1 expression may influence renal iron excretion rate. A recent *in vivo* study showed that altered dietary iron intake is a strong modulator of renal DMT1 expression in male Wistar rats (Wareing et al., 2003). Increasing dietary iron (an iron-enriched diet, 5 g/kg) was found to decrease DMT1 expression and increase urinary iron excretion rate, while decreasing dietary iron (an iron-restricted diet, 50 mg/kg) caused an increase in DMT1 expression and a decrease in urinary iron excretion (Wareing et al., 2003). This finding is different from what was found in the present study, where we found FAC to have no effect on DMT1. The cause of this conflict in results is unknown, although it may be partly due to the differences between experimental conditions *in vivo* and *in vitro*.

DMT1 is found not only in proximal tubule cells, but also in late thick ascending limbs, early distal tubules, and intercalated cells of the cortical collecting duct (Ferguson et al., 2001; Abouhamed et al., 2006). The types of epithelial cells found at different segments of nephron are different, which is why different segments have different functions in re-absorption and secretion for practical ions such as Na+ and K+ (Silverthorn, 2001). Currently it is unknown whether iron handling differs between different segments of the nephron. It is also unknown whether the responses of DMT1 to iron differ between different parts of nephron, although it is certainly possible. In addition, it has yet to be determined whether iron within the peritubular capillaries can be moved into the interstitial space, then to tubular cells and finally, be deposited into tubular fluid, especially when there is an increase in serum iron. Furthermore, the role of L-type Ca2+ channels in iron transport in different segments of the nephron still need to be clarified. Only after we have the answers to the above questions, can we fully account for how nifedipine stimulates urinary iron excretion in the kidney.

## Author Contributions

YK and Z-MQ conceived, organized and supervised the study; S-SY, YL, Y-FZ, JL and L-RJ performed the experiments; YK and Z-MQ contributed to the analysis of data, and prepared and wrote the manuscript.

## Conflict of Interest Statement

The authors declare that the research was conducted in the absence of any commercial or financial relationships that could be construed as a potential conflict of interest.

## Abbreviations

BSA: bovine serum albumin
DMT1: divalent metal transporter1
DMT1 + IRE: divalent metal transporter1 with iron response element
DMT1 - IRE: divalent metal transporter1 without iron response element
FAC: ferric ammonium citrate
Fpn1: ferroportin1
Tf: transferrin
TfR1: transferrin receptor 1
MTT: 3-(4,5-dimethylthiazol-2-yl)-2,5-diphenylte-trazolium bromide
